# Relationship between Residency Burnout and Suicidal Risk in the Resident Physician Population

**DOI:** 10.1192/j.eurpsy.2023.713

**Published:** 2023-07-19

**Authors:** S. Jacob, R. Tabraiz, H. Raai

**Affiliations:** St. Barnabas Hospital, Bronx, United States

## Abstract

**Introduction:**

Resident physicians compared to the general public are exposed to a more rigorous schedule. Burnout as described by the World Health Organization is a phenomenon occurring in an occupational setting. It consists of three domains: feelings of exhaustion, reduced professional efficacy, increased mental distance from one’s own job. Research shows that increased working hours are associated with higher levels of burnout in resident physicians.

**Objectives:**

Through literature review we will explore whether this burnout contributes to an increased suicidal risk in the resident physician population.

**Methods:**

Various studies assessing training of residents globally were analyzed and compared. A study in Japan distributed a survey to 4306 resident physicians. Suicidal ideation was noted in 5.6% of these physicians but when working more than 100 hours in the hospital the rate increased to 7.8%. In Australia it was found that once doctors in training worked more than 55 hours per week there was was an increase of 50% in suicidal ideation. It was also found that 12.3% of the people surveyed in the Australian study had reported suicidal ideation within the past 12 months of the survey. A study observing 5126 Dutch residents found 12% of residents having suicidal ideation but double in the group with burnout vs the group without burnout.

**Results:**

The studies listed show that increased work hours and burnout was associated with increased suicidal ideation in medical residents. A study observing 1354 physicians in the US found that higher measurements of burnout were associated with suicidal ideation similar to previous studies. However once adjusted for depression, it was noted that there was an association with depression and suicidal ideation but not with burnout. Depression may be a confounding variable that may have not been adjusted for when determining the association of burnout with suicidal ideation. In addition further research looking at the leading cause of death among a total of 381,614 US medical residents between the years 2000 to 2014 found suicide as the second most common cause of death. It was however found when looking at resident physicians between the age of 25-34.9 there was 4.07 suicides per 100,000 person years while in the general public there was 13.07 suicides per 100,000 years.

**Conclusions:**

The rate of suicide was found to be lower in resident physicians compared to the general public. Suicidal ideation may be more closely associated with depression versus burnout itself and should be accounted for when assessing suicidal ideation in the resident physician population. Suicide rates being lower in resident physicians compared to the general public bring up the possibility that burnout in resident physicians does not have to be directly correlated with increased risk of suicide.

**Disclosure of Interest:**

None Declared

